# Information and communication technology development and health gap nexus in Africa

**DOI:** 10.3389/fpubh.2023.1145564

**Published:** 2023-03-30

**Authors:** Ebenezer Toyin Megbowon, Oladipo Olalekan David

**Affiliations:** School of Economic Science, North-West University, Vanderbijlpark, South Africa

**Keywords:** ICT development, life expectancy (LE) at birth, international agreement, Alma Ata Declaration, health gap, Africa

## Abstract

**Introduction:**

Development of information and communication technology has been identified as a tool for fast and effective information gathering and dissemination, and as a means through which almost every social and economic sector (including the health sector) could achieve economic, operational, and service delivery efficiencies that can enable the realization of targeted outcomes. ICT can serve as a tool for achieving international agreements (including the Alma Ata Declaration of 1978), thereby accelerating the achievement of various global development targets.

**Methods:**

Consequently, based on a sample of 38 countries from 2000 to 2018, this study investigates the effect of ICT development on the health gap, and whether the effect varies by gender and sub-region in Africa. The dependent variable (health gap) was measured as the difference between the achieved life expectancy at birth of 60 years and the Alma Ata Declaration of 1978 targeted life expectancy at birth of 60 years. The main independent variables are ICT indicators (ICT index, mobile cellular subscriptions, and internet access), while the gross domestic product (GDP), which is the measure of economic growth, healthcare expenditure, urbanization, and labor market outcome, is employed as control variables. The effect was examined using Driscoll-Kraay standard errors, feasible generalized least squares (FGLS), and panel-corrected standard error (PCSE).

**Results and discussion:**

The findings of the Driscoll-Kraay standard errors estimation technique supported by those of FGLS and PCSE suggest that ICT does act as an indispensable stimulator for Africa to significantly exceed the international health target of life expectancy at birth of 60 years. It can be concluded that African leaders need to take advantage and maximize the health-enhancing potential of the internet component of ICT through relevant policies that would improve internet coverage, connectivity, and access for individuals and health institutions.

## Introduction

The importance of the health of a population, as well as the health system of a country, cannot be over-emphasized. Health, as a component of human capital, is one of the identified key factors that determine growth in the economic activities of a country through a healthy labor force. At the individual level, health affects labor participation, productivity, income, and welfare in general. The importance of health is heightened in its inclusion as the third objective in the sustainable development goals (SDGs), which state that ensuring healthy lives and promoting wellbeing at all ages is essential to sustainable development (1). The distribution of and access to basic health services across the social spectrum of a country as well as the quality of health of the population is part of the preconditions for economic and social development (2). Health indicators are directly related to the health quality, health systems, and development of countries (3). These indicators act as measures of the performance of healthcare systems and the wellbeing of the population (4). The COVID-19 pandemic that destabilized the global economy, toppled the lives of billions of people around the globe, and increased human suffering as noted by the UN (5) further emphasizes the indispensability of health in economic development and the imperativeness of healthcare delivery improvement. The evaluation of health system performance and progress toward stated goals for a given society in the pursuit of healthcare delivery improvement necessitates the monitoring of changes in health status over time (6), access channels, alternatives, and opportunities for possible health delivery improvement.

Otto et al. (7) noted that traditional approaches to improving basic healthcare delivery are giving way to a paradigm shift toward modern approaches seen in fast and efficient information flow and improved medical infrastructures including a view and incorporation of digital provide—information and communication technology (ICT). This is because information in all its forms, for adequate and effective decision-making, is one of the features of the twenty first century, and ICT as a technological innovation provides means of easy exchange and convey of information without limitations (spatial or temporal barriers) at an efficient level with minimal cost implications (8). ICTs embody all digital technologies that support the electronic capture, storage, processing, and exchange of information, such as fixed-line telephones, mobile phones, the internet, and broadband (9). The world has rapidly advanced in the direction of applied economics—healthcare and a general economic system based on continuous and widespread innovations that heavily rely on ICT (10, 11). Khan et al. (12) buttressed that ICT has revolutionized societies around the world and is incorporated into almost all activities of human life including economic growth, education, agriculture, finance, governance (13), international trade, environment, and disaster management among others. ICT is documented to be equally applicable to every component of the healthcare sector, and its relevance is ultimately in its potential to directly reduce communication gaps between health experts when engaging in their profession, indirectly improve a healthy society, and as well reduce mortality rates. This is possible with ICTs' direct link with (i) health monitoring/tracking and diagnostics, (ii) medical supplies, (iii) access to doctors, (iv) health information dissemination, and (v) health data management.

In terms of health information dissemination, management, tracking, and diagnostics, ICT creates platforms for health-oriented technology solutions such as electronic medical information recording and retrieval, short message service (SMS), health data monitoring, diagnosis, and long-distance treatment (14). Through SMS, pregnant women and children are reliably informed or reminded of their necessary hospital check-ups and visit. The information-oriented nature of patient health management has helped in the development of devices for self-use and easy monitoring of chronic diseases such as diabetes. The internet can facilitate the provision of health services for expectant and nursing mothers and interaction between a patient and respective health systems (15, 16). Qiang et al. (17) summarize that ICT in health through mobile health (mHealth) facilitates health quality and access through treatment support, patient tracking, supply management, health financing, and emergency services. In terms of medical supplies, ICT tools have been used for healthcare supply chain management, such that there is a reduction in delay periods for medicine shipments and provision of point-of-use technologies for consumers to verify the authenticity of medical products that is being bought and report of shortages of medical supplies using SMS. Also, ICT in healthcare delivery is seen in the areas of access to doctors through virtual healthcare (VH). VH is considered a model of health service delivery, which substitutes in-person consultations with telephonic or video consultations (18). Compared to direct-person care in an office space, VH enhances convenient, easy, and faster access to healthcare service providers and specialists in a cost-efficient manner (19). The sum of the effects of the stated theoretical explanations of the ICT-health dynamics nexus is improved health status, access to quality health, reduced mortality rate, and consequently an increased life expectancy.

In Africa, several countries have ICT initiatives and solutions that have been deployed in their health system. For example, Health Management Information System (HMIS) for electronic medical recording (EMR) is being used in Ghana, Rwanda, Kenya, South Africa, Ghana, Lesotho, Zimbabwe, Zambia, Uganda, Tanzania, Mozambique, Morocco, and Tunisia. Telemedicine is also used in South Africa, Nigeria, Ghana, Ethiopia, Morocco, and Tunisia (20). Olu et al. (21) also highlighted the use of computer-aided detection of tuberculosis by chest x-ray has been used in Zambia, South Africa, and The Gambia, and digital ultrasound (using mHealth/telemedicine solutions) has been utilized in Tanzania. Rapid diagnostic tests (RDTs) integrated into cloud-based mHealth smart reader systems are in use in Kenya, Tanzania, and Ghana, while smartphone-powered, cloud-enabled portable electrocardiographs (ECGs) are used in Uganda and Malawi. Similarly, ICT interventions through mobile SMS have been used as a reminder for antenatal and postnatal care attendance in Tanzania and South Africa, respectively. Indeed, in agreement with Olu et al. (21), considering all the possibilities embedded in ICT, ICT though not an end, is no doubt a means to an end.

Equally, the relevance and importance of ICT in healthcare digitization and disease surveillance in the period of epidemics or pandemics cannot be understated. It was observed that there was increased use of electronic resources amidst lockdowns and social distancing during the COVID-19 pandemic. Several governments of nations through adopting e-governance created a robust, efficient, and reliable network of communication between the government and the people (22). Similarly, ICT tools such as tracking apps were deployed by governments to monitor the spread of the pandemic by monitoring infected persons and tracing their contacts (23). There were digital tools such as smart infection detectors, 5G technology, telemedicine system, drones, robots, and digital quick response (QR) codes and automated vehicles that were deployed for efficient and fast COVID-19 diagnosis, intra and inter-border COVID-19 test results transmission, dissemination of COVID-19 statistics, awareness, cautions, spreading of control measures, enabling medical service providers to work remotely or practice self-isolation measures, disinfecting cities, delivering goods (24, 25). Similarly, there was high-level international coordination and collaboration between government–government and development partners about sharing national situation updates on COVID-19 and distribution of vaccines that were facilitated through video- and audio-conferencing tools. ICT tool utilization during the COVID-19 crisis as evident in digital health and e-governance contributed to improvement in institutional quality and performance, improved quality of life, helped people save time, and improved accessibility of public services (22). Considering the dynamic importance of digital tools, the G20 recognizes digital health as a fundamental tool, especially during this pandemic, and has committed to placing digital health as a key element of health services infrastructure and a key strategy to improve value in healthcare service delivery at the G20 Riyadh Summit in 2020. Furthermore, the Riyadh (2020) Summit emphasized the critical importance of the country's “building blocks,” including but not limited to the readiness of IT infrastructure, equity of access, and cost-effective services, as well as workforce and institutional capacity, in achieving digital health success (26).

Improved life expectancy at birth is an end that this study posited to be achievable through ICT. The importance attached to increasing life expectancy at birth is seen in various international conventions and agreements that have been entered into by governments of countries of the world. These include “The Declaration of Alma Ata, 1978” which set a target of life expectancy >60 years by the year 2000; the World Summit for Social Development (WSSD), Copenhagen 1995 which also adopted the Declaration of Alma Ata, 1978 goal. The International Conference on Population and Development (ICPD) Programme of Action specified that life expectancy should be > 65 years by 2005 and 70 years by 2015 for countries that currently have the highest levels of mortality; and 70 years and 75 years, respectively, for the other countries (ICPD Programme of Action, paragraph 8.5) (27–30). Life expectancy at birth is an important measure of health status (31) and a health indicator that reflects the condition of the health system of countries. Increased life expectancy at birth reflects an improved health system, which stimulates an increase in the number of healthy people and the labor force, with implications for individuals and the wealth of a nation. An increase in life expectancy at birth increases the stock of human capital and manpower needed for economic productivity and growth, mostly through more long-term investment in education and greater accumulation of knowledge (32, 33). Similarly, an increased life expectancy at birth extends the labor participation timeframe, resulting in an increase in retirement age. This consequently enables physical capital accumulation through saving (34). Hence, it can be said that besides the many effects of the COVID-19 pandemic, healthcare service delivery improvement is further important for achieving healthcare targets.

The health gap in this study is conceptualized as a measure of variation between actual health outcomes and preferred or targeted health outcomes. This is in line with Murray et al. (6), who identified the health gap as one of the summary measures of population health and defined it as the measure of the difference between the current health condition of a population and a selected target. Health gap though could be dynamically conceptualized but has been majorly centered on healthy life year (HeaLY), quality-adjusted life year (QALY), and disability-adjusted life year (DALY). The simple conceptualization of the health gap in this study provides a clear understanding of monitoring changes in health status over time and evaluating health system performance and progress toward achieving definite goals. Based on the premise of this study as earlier mentioned, it is posited that ICT has the potential to aid countries' achievement of health targets. However, the contribution of ICT or the extent to which ICT can contribute to health target achievement (The Declaration of Alma Ata, 1978 life expectancy target) needs to be evaluated. Hence, the objective of this study, therefore, is to access the effects of ICT on the health gap. The specific objectives are as follows: (i) to examine the level of ICT development, (ii) to profile the health gap and the extent of the gap, and (iii) to estimate the effect of ICT on the health gap. This study makes two important contributions to the literature, first by filling a knowledge gap by analyzing international agreement (The Declaration of Alma Ata, 1978) target vs. achieved outcome gap within the African context. Apparently, this gap analysis is imperative to examine the commitment of countries to international agreements. Second, the study filled a methodological gap through the utilization of the gap variable which is a novel health outcome variable that is different from what has been used in previous related studies to provide new insight into the effect of ICT on health outcomes. The rest of the study is structured as follows: Section Literature review reviews relevant theoretical frameworks and empirical literature, and the analytical techniques and data utilized in this study are outlined in Section Methodology. Discussion and conclusion with relevant policy recommendations are presented in Sections Empirical results and analysis, Conclusion and policy recommendations, respectively.

## Literature review

### ICT and development-health outcomes relationship: Theory

The link between ICT and development outcomes, in general, has been explained by different theories in the literature. The fundamental role played by technological change and innovation is embedded in Schumpeter's theory of innovation, entrepreneurship, and economic development. According to Hartmann (35), Schumpeter (36), Schumpeter (10) illustrated development as a historical process of structural and qualitative changes that are mostly driven by innovation. This innovation process which has four dimensions, i.e., inventions, innovation, diffusion, and imitation, is defined as a new combination leading to new products, processes, organizations, inputs, and markets (35). Also, Verspagen (37) noted that the historical role of pervasive technologies has been the quiet subject of Schumpeter's concept of economic growth and development and alluded that structural change, economic growth, and major technological innovations and breakthroughs are closely interconnected. The pervasive nature of these technological innovations further underscores the relevance of Schumpeter's thought on the relationship between ICT and development. ICT considered general-purpose technology (GPT) as propounded by Bresnahan and Trajtenberg (38) because it has impacts across several sectors of an economy owing to that it has been used as inputs and has facilitated new ways of engagement or of doing things more efficiently and effectively. Such that with ICT, more organizational opportunities have been created, performance (such as productivity and expansion) and services have been enhanced, the cost has been reduced, and revenue has been increased.

Similarly, another relevant theory on ICT-health linkage is the determinant of demand for health, health production, and demand for medical care embedded in the seminal work of Grossman (39), which is inspired or premised on the theory of human capital of (40). It is conceptualized in the human capital theory that a stock of human capital raises an individual's productivity. The theory basically motivates the need for investment in education through on-the-job training or formal education to realize desired productivity gains. Grossman (39), however, through his seminal work on a human capital model of demand for health differentiated between human capital and health capital and argued that health capital stock has some level of influence on both non-market and market-related productivity efficiencies of a person. According to Grossman (39), health is first conceived as a capital good and a consumption good that yields satisfaction both indirectly (through lesser days of illness, increased productivity, increased wages) and directly, and then as an output that is influenced by various inputs. Health in the Grossman model is seen as an investment in human capital as well as an output in the household production process (41). Thus, through this conceptualization, demand for health and demand for medical services is put forward. Wagstaff (42) further explained that the health production function relating to the determinant of health demand in Grossman's theory is based on the classical production idea, where output is a function of the combination of several inputs, and in this case, the output is health outcome, and inputs include demographic, geographic, environmental, economic factors, and innovation or technological change, respectively.

### ICT-health outcome relationship: Empirics

The global advancement in ICT and its deployment in the past 30 years has continued to attract increasing attention among development partners and academics who have focused on investigating the dynamic impact of ICT diffusion on development outcomes including health in both developed and developing countries, respectively. An interesting strand of literature documents the development outcomes impact of ICT in terms of the human development index (11, 43), economic growth (44–46), corruption (46), and financial inclusion (47, 48) among others. However, opposing results which are often attributable to the scope of the study and the econometric methodologies adopted have been observed.

In terms of health outcomes, a very large number of studies (3, 31, 49–65) have been undertaken to examine the determinants of various health outcomes outside ICT as a determining factor. In recent times, there is growing empirical literature investigating the connection between ICT and health outcomes that has been undertaken at the macro level. Kouton et al. (66) employed a two-step system generalized method of moments (GMMs) over a panel of 35 countries in Sub-Saharan Africa over the period 2000–2016 to investigate the health outcome (under-five mortality) impact of ICT. The ICT development index was constructed from a principal component analysis (PCA) of the number of individuals with access to the internet and the mobile penetration rate. The study found that ICT has a negative and significant effect on under-five mortality at the 5% level of significance, implying that ICT development significantly reduces under-five deaths. Dutta et al. (67) measured the ICT index with the PCA technique that incorporates secure internet servers, internet access, fixed broadband subscriptions, fixed telephone subscriptions, and mobile cellular subscriptions as drivers of infant mortality rate. The study sampled a panel of 25 Asian countries using both fully modified ordinary least squares (FMOLS) and dynamic ordinary least squares (DOLS) panel estimators and revealed that ICT has a significant negative relationship with infant mortality rate. Meaning that health outcome proxy by infant mortality rate will decrease as a result of an increase in the ICT infrastructure.

Dutta et al. (2) evaluated the effect of mobile phone penetration on health outcomes using cross-country data from 25 Asian countries from 2000 to 2014, with infant deaths (before completing 1 year) per 1000 live births used as a proxy for health outcome. The study demonstrated that mobile phone penetration using FMOLS and DOLS has the potential to improve the health outcome of the countries under-examined following a computed negative coefficient and significant value of mobile phone penetration. Majeed and Khan (68) analyzed the relationship between ICT and health outcome proxy by life expectancy at birth and infant mortality rates among, 184 countries for the period 1990-2014 using two-stage least squares and system GMM. The empirical result showed a positive and significant impact of ICT on population health, though the extent of the impact is very minute.

Bankole and Mimbi (69) investigated the impact of ICT infrastructure on health systems proxy by life expectancy at birth, infant mortality rate, health expenditure per capita, and health expenditure [as % of gross domestic product (GDP)] in Africa, using partial least squares on data from 27 African countries for the period 1998–2007. The findings indicate that ICT significantly improves life expectancy at birth and reduces infant mortality rates, respectively. However, ICT was also found to have an increasing effect on healthcare expenditures. Adeola and Evans (70) examined the relationship and causality between ICT and health expenditure for 49 African countries for the period 1995–2015 using the panel GMM model and Toda-Yamamoto causality tests. The empirical results show that ICT has a positive and statistically significant relationship with health, suggesting that the increase in ICT in the region, positively stimulates the level of health. A bi-directional causality between ICT and health was also established.

A study conducted in Ghana by (71) provided evidence that supports the argument that ICTs influence the demand for reproductive health services. Specifically, the coefficients of mobile phone ownership obtained from a probit regression estimate were found to be consistently positive and significant across all four reproductive health services examined (i.e., current use of contraception, the timing of first antenatal check-up, place of delivery, and antenatal care). The study argued that women who reside in households where these ICT commodities are present will have a relative advantage over their counterparts in households without with respect to maternal healthcare services utilization. Afroz et al. (72) established from an ARDL estimation that while mobile cellular subscriptions reduce infant mortality in Malaysia, fixed broadband subscriptions of ICT performed otherwise.

Through ordinary linear regression estimations, Tavares (73) showed that ICT development and adoption of e-health do not have a significant relationship with health status and reporting of unmet health needs. It was, however, found that ICT development has a significant impact on self-reported chronic health challenge reporting among European Union (EU) countries examined. In the study, an increase in ICT reporting increases with advancement in the development of ICT. Irawan and Koesoema (74) support findings from previous studies by establishing that a lower level of ICT development correlates with increased maternal mortality and child mortality, respectively. The effect of ICT on health outcomes from previous studies appears to be consistent; however, the effect of ICT in relation to meeting health targets and the extent of meeting the target seems not to have been considered. Thus, in contributing to the discussion of ICT and health, this study examined the impact of ICT in relation to the health outcome target of life expectancy at birth. This study contributes to the literature by introducing a novel outcome (health gap) and attempting to access the extent of achievement of internationally agreed Alma Ata (1978) declarations in the African region. A gap analysis as part of health performance assessment helps clarify the difference between reality (actual outcome) and the ideal (a desirable outcome), which enables better utilization of resources to close or improve the target-actual outcome gap.

## Methodology

### Theoretical framework and model specification

The study is premised on the Grossman health production function and Schumpeter's theory of structural change as discussed in the literature review section. While Grossman's health production function reflects how much health can be achieved with respect to available health inputs (e.g., health expenditure, education, urbanization, income, environmental degradation, food security, alcohol consumption, health facilities, and population), Schumpeter, on the other hand, facilitates the idea of innovation (e.g., ICT) as an indispensable input in the development process which thus include health progress. Therefore, in line with the two theories, the mathematical functional model for this study is presented in Equation (1);


(1) $$\begin{array}{l} HG=f\left(HI\right)\ldots \ldots \ldots \ldots \ldots \ldots \ldots \ldots \ldots .. \end{array}$$

where HG represents the health gap and health outcome and HI represents a vector of health inputs. After incorporating necessary health inputs including ICT, the extended functional representation of the effect of ICT along other health inputs in the health gap equation in Equation (1) can be written as follows:


(2) $$\begin{array}{l} HG=f\left(ICT,\;Y,\;PuH,\;PrH,\;U,\;E\right)\ldots \ldots \ldots \ldots \ldots \ldots \end{array}$$

Equation (2) can be expressed in Cobb–Douglas production form, considering HG as health output being determined with other health inputs.


(3) $$\begin{array}{l} HG=\;{\left(ICT\right)}^{\beta_{1}}\;{\left(Y\right)}^{\beta_{2}}\;{\left(PuH\right)}^{\beta_{3}}\;{\left(PrH\right)}^{\beta_{4}}\;{\left(U\right)}^{\beta_{5}}\;{\left(E\right)}^{\beta_{6}}\ldots \ldots \ldots \end{array}$$

The Cobb–Douglas's Health Production Function in Equation (3) is further semi-linearized by applying natural logarithms to the dependent variables thus giving a linear-log Cobb–Douglas health production function. This can be written as follows:


(4) $$\begin{array}{l} HG_{it}=\beta_{1}lnICT_{it}+\beta_{2}lnY_{it}+\beta_{3}lnPuH_{it}+\beta_{4}lnPrH_{it}+\beta_{5}lnU_{it}\\ \;+\beta_{6}lnE_{it}+\varepsilon_{it}\ldots \ldots .. \end{array}\;\left(4\right)$$

where I = countries 1, 2, 3 …38, t = period 2000 to 2018, HG is health gap, ICT is information and computer technology, Y is economic growth, PuH is public expenditure on health, PrH is private expenditure on health, U is urbanization, and E is employment. To estimate the responsiveness of the health gap to the variations in ICT and other identified variables, a static panel model estimation technique (Driscoll–Kraay standard errors) was utilized. Since panel data are often faced with problems of serial correlation, heteroscedasticity, and cross-sectional dependence, the Driscoll–Kraay standard errors technique was used because its estimation is robust to general forms of cross-sectional and temporal dependence, and the errors generated are heteroscedasticity and autocorrelation consistent. Furthermore, to check for the robustness of the main model and estimation technique that was employed, the estimation can be conducted by using related variables or employing alternative estimation techniques. Thus, in this study, robustness was checked by disaggregating ICT as well as by using other estimation techniques, including feasible generalized least squares (FGLS) and panel-corrected standard error (PCSE). Both techniques provide estimates that put the problems of unobserved autocorrelation and heteroscedasticity into consideration, thus producing robust standard errors (75–77).

### Data

This study compiled and utilized data on life expectancy at birth (used for computing health gap), ICT index (computed through PCA from a combination of mobile cellular subscriptions and internet access), gross domestic product, public healthcare expenditure, private healthcare expenditure, urbanization, and employment for a sample of 38 economies in Africa. The data utilized in the study were sourced from the World Bank listed in Table A1, World Development Indicator, and International Telecommunication Union (ITU) websites. All data were converted to natural logarithms except the health gap variable and they cover the time from 2000 to 2018 for all sampled countries.

### Description of variables

In line with similar studies, the main variables are the health gap and the ICT index. For robustness, five control variables, gross domestic product (GDP) which is the measure of economic growth, healthcare expenditure, urbanization, and employment, these indicators are explained in brief in the following paragraphs.

Health gap (HG) is a health outcome and the main variable of interest in this study. The variable is measured by finding the difference between achieved life expectancy at birth (LEB) and life expectancy at birth target. The variable is measured in years.


(5) $$\begin{array}{l} HG=LEB\;achieved-LEB\;target\;\left(60\;years\right) \end{array}$$

ICT is the main independent variable of interest. Two proxies (mobile cellular subscriptions (per 100) and population internet access (individuals using the internet as % of the population) were used in this study to generate an ICT index through principal component analysis (PCA). Following empirical findings reported in the literature review section, it is posited in this study that there will be a health gap-enhancing effect from ICT. Thus, the ICT variable is expected to have a positive coefficient.

Economic growth (Y) is generally agreed to be a necessary condition for development. With improvement in economic activities, a country is enabled to provide necessary infrastructure and expenditure that could aid wellbeing, such as healthcare infrastructure, increase in health research, and provide resources to combat any health-related challenges. Another indirect effect of economic growth on health outcomes is the access of the population to adequate nutrition and decent housing through an increase in per capita income (68), which altogether stimulates health outcomes. Therefore, it is expected that the economic growth variable in this study would have a positive relationship with the health gap. This variable is proxied by GDP and measured in constant 2010 US$.

Healthcare expenditure is the amount spent by both individuals and the government on healthcare. It is measured as an out-of-pocket expenditure (% of current health expenditure) for private and domestic general government health expenditure (% of GDP) for the public sector. An empirical finding by Al-Azri et al. (78) show that both public and private healthcare expenditure have significant positive effects on the health outcome of life expectancy. While government spending on health facilitates health sector investment and necessary awareness, private healthcare expenditure relates to access to health services. In this study, private health expenditure is represented as PrH, while public sector expenditure is represented as PuH.

Urbanization (U) is a geographical location-related variable. The impact of urbanization on health generally has been mixed such that it depends on the level of planning of the urban sector. While unplanned rapid urbanization negatively affects health situations through poor housing conditions, overcrowding, and poor environmental sanitation that stimulate disease spread, well-planned urban structure improves the health system through better access to education, infrastructural facilities, health facilities, and better access to necessary health improving information (79, 80). Based on the reported mixed effect of urbanization, the study hypothesized that the effect of urbanization on the health gap would be either negative or positive. This variable is proxy by the urbanization growth rate, and it is measured in percentage.

Employment (E) in this study is proxy by labor force participation rate which is defined as the proportion of the population in the labor force age group (aged 15 and older) that is economically active, and it is measured in percentage. According to Goodman (81), employment has social, psychological, and financial benefits that improve health. The effect of employment on health outcomes, however, depends on the quality of work and the sector of work. For instance, labor market participation in a formal sector has the tendency to improve health outcomes since formal sector employment makes provision for health improving resources such as health insurance. Goodman (81) further explained that well-paying work provides individuals with the financial means to access nutritious food, healthcare, and safe housing, all of which have an impact on health directly.

## Empirical results and analysis

### Stylized facts and trend

This section first presents and discussed trends in ICT development followed by an assessment of the health gap in Africa.

### ICT development

**Figure 1:**
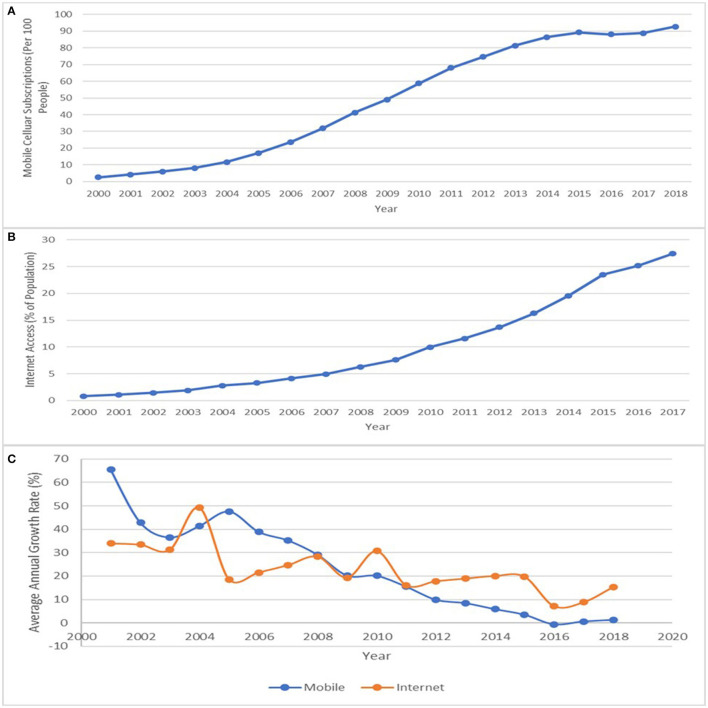
**(A)** Mobile cellular subscription, **(B)** internet access, and **(C)** average annual growth rate in mobile cellular subscription and internet access (2000–2018). Source: Data for the Graph was sourced from ITU.

**Figure 2:**
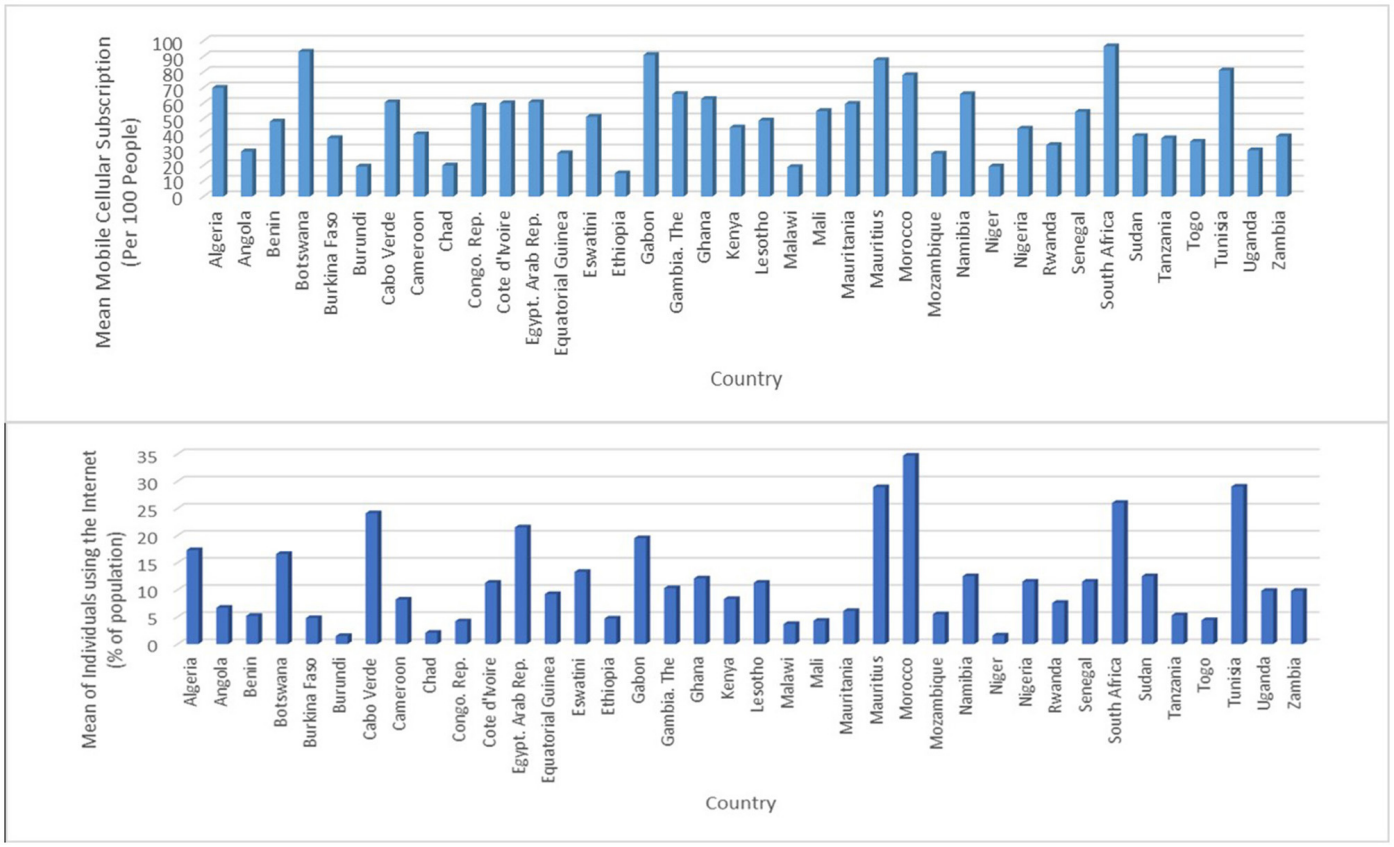
Average mobile phone subscription and internet access by country (2000–2018). Source: Data for the Graph was sourced from ITU.

A graphical representation of ICT development across the African region is first presented then followed by country-level performance. The trend in ICT development in Africa is shown in Figure 1, while a comparative country-level performance is shown in Figure 2 and Table 1, respectively. Figure 1A showing the level of mobile cellular subscriptions among the selected Africans from 2000 to 2018, clearly indicates that there has been a continuous growth in mobile cellular subscriptions in the region. This is a reflection that mobile phone penetration has been immense. Figure 1A reveals that average mobile telephone penetration was at its lowest in the year 2000 with an index of 2.57 but increased to 17.29 in 2005. The average index of mobile telephone penetration further increased to 92.49 in 2018. Figure 1B shows the trend of average internet access across time considered among the selected African countries.

**Table 1:** Ranking of ICT development at 2018.

**Ranking**	**Country**	**Mobile**	**Country**	**Internet**	**Country**	**ICT Index**
1	South Africa	159, 9	Morocco	64, 8	South Africa	157.2
2	Mauritius	151, 4	Tunisia	64, 2	Mauritius	148.5
3	Botswana	150, 0	Mauritius	62, 4	Botswana	147.1
4	Gambia. The	139, 5	South Africa	59, 6	Gabon	139.9
5	Gabon	138, 3	Cabo Verde	59, 5	Tunisia	135.7
6	Ghana	137, 5	Egypt. Arab Rep.	58, 6	Morocco	133.6
7	Cote d'Ivoire	134, 9	Gabon	58, 0	Ghana	127.6
8	Tunisia	127, 7	Algeria	49, 0	Gambia. The	126.9
9	Morocco	124, 2	Botswana	48, 0	Cote d'Ivoire	121.9
10	Mali	115, 1	Eswatini	46, 9	Cabo Verde	121.4
11	Namibia	112, 7	Namibia	43, 0	Algeria	113.6
12	Cabo Verde	112, 2	Sudan	40, 0	Namibia	108.0
13	Algeria	111, 7	Ghana	40, 0	Egypt. Arab Rep.	100.6
14	Senegal	104, 5	Nigeria	37, 5	Senegal	98.8
15	Mauritania	103, 7	Senegal	35, 3	Mali	97.6
16	Burkina Faso	97, 9	Lesotho	35, 0	Mauritania	88.2
17	Kenya	96, 3	Cote d'Ivoire	31, 9	Eswatini	88.1
18	Congo. Rep.	95, 3	Gambia. The	31, 1	Lesotho	87.0
19	Egypt. Arab Rep.	95, 3	Uganda	31, 0	Nigeria	84.9
20	Eswatini	93, 6	Zambia	29, 7	Kenya	81.9
21	Zambia	89, 2	Equatorial Guinea	27, 0	Burkina Faso	79.1
22	Nigeria	88, 2	Kenya	26, 0	Benin	76.6
23	Benin	82, 4	Cameroon	25, 0	Congo. Rep.	73.8
24	Rwanda	78, 9	Rwanda	24, 0	Rwanda	73.4
25	Togo	77, 9	Angola	23, 0	Zambia	73.2
26	Tanzania	77, 2	Mauritania	22, 0	Sudan	72.9
27	Lesotho	75, 0	Mozambique	21, 0	Cameroon	72.8
28	Cameroon	73, 2	Tanzania	19, 5	Tanzania	68.1
29	Sudan	72, 0	Benin	19, 0	Togo	66.0
30	Uganda	57, 3	Burkina Faso	15, 5	Uganda	57.5
31	Burundi	56, 5	Ethiopia	14, 3	Angola	55.2
32	Mozambique	47, 7	Togo	14, 0	Equatorial Guinea	51.0
33	Equatorial Guinea	45, 2	Mali	13, 9	Burundi	43.5
34	Chad	45, 1	Congo. Rep.	10, 4	Ethiopia	43.3
35	Angola	43, 1	Malawi	9, 0	Mozambique	41.1
36	Niger	40, 7	Chad	8, 0	Chad	37.6
37	Ethiopia	39, 2	Niger	5, 3	Malawi	37.4
38	Malawi	39, 0	Burundi	5, 0	Niger	32.5
Average 2018		92, 9		32.3		88.5
Average 2000-2018		49.7		11.5		43.5

Source: Data used in ICT development ranking was obtained from ITU.

The results show that internet access stood at 0.81 in 2000 and increased to 4.12 in the year 2006. The increasing trend in mean internet access increased from 10.28 in 2010 to 32.3 in 2018. While there is an increase, however, there is an observable decline in the rates of growth of both ICT components, which is more visible in the case of mobile cellular subscription, as shown in Figure 1C.

The descriptive comparative performance analysis in ICT development by country is shown in Figure 2 and Table 2, respectively. Figure 2 shows the diversity in the mobile cellular subscriptions among countries examined when looking at the average across the years considered. The figure reveals that South Africa at 96.49 had the highest mean mobile cellular subscriptions, followed by Botswana (92.99), Gabon (90.94), Mauritius (87.59), Tunisia (81.1), Morocco (78.03), and Algeria (69.83), respectively. Equally, within the period under consideration, mobile communication penetration is low in Rwanda (33.01), Uganda (29.76), Angola (28.88), Equatorial Guinea (27.80), Mozambique (27.47), Chad (19.87), Niger (19.28), Burundi (19.18), Malawi (18.9), and Ethiopia (14.85), respectively. Though an increase in mobile cellular subscriptions is observed in 2018, as shown in Table 2, 18 countries out of 38 countries examined had a mobile cellular subscription that is less than the 2018 average, whereas seven countries (Mozambique, Equatorial Guinea, Chad, Angola, Niger, Ethiopia, and Malawi) had <2000–2018 average mobile phone subscription. The leading countries with mobile phone penetration as of 2018 are South Africa, Mauritius, Botswana, The Gambia, and Gabon.

**Table 2:** 2018 health gap ranking by total and gender.

**Ranking**	**Country**	**Health Gap (Total)**	**Country**	**Health Gap (Female)**	**Country**	**Health Gap (Male)**
1	Algeria	16.69	Tunisia	18.54	Algeria	15.49
2	Tunisia	16.51	Algeria	17.94	Morocco	15.19
3	Morocco	16.45	Mauritius	17.72	Tunisia	14.49
4	Mauritius	14.42	Morocco	17.67	Mauritius	11.27
5	Cabo Verde	12.78	Cabo Verde	16.01	Egypt. Arab Rep.	9.60
6	Egypt. Arab Rep.	11.83	Egypt. Arab Rep.	14.16	Cabo Verde	9.32
7	Botswana	9.28	Botswana	12.05	Rwanda	6.52
8	Rwanda	8.70	Rwanda	10.78	Botswana	6.20
9	Senegal	7.67	Senegal	9.63	Senegal	5.50
10	Kenya	6.34	Kenya	8.68	Ethiopia	4.35
11	Ethiopia	6.24	Gabon	8.32	Gabon	4.15
12	Gabon	6.19	Ethiopia	8.17	Kenya	3.97
13	Sudan	5.10	South Africa	7.40	Sudan	3.28
14	Tanzania	5.02	Sudan	6.95	Tanzania	3.17
15	Mauritania	4.70	Malawi	6.94	Mauritania	3.08
16	Congo. Rep.	4.29	Tanzania	6.82	Congo. Rep.	2.84
17	South Africa	3.86	Zambia	6.45	Ghana	2.72
18	Malawi	3.80	Mauritania	6.29	Niger	0.90
19	Ghana	3.78	Namibia	6.18	Uganda	0.66
20	Zambia	3.51	Congo. Rep.	5.70	Malawi	0.65
21	Namibia	3.37	Uganda	5.17	Zambia	0.53
22	Uganda	2.97	Ghana	4.85	South Africa	0.46
23	Niger	2.02	Eswatini	3.97	Gambia. The	0.36
24	Gambia. The	1.74	Angola	3.67	Burkina Faso	0.36
25	Benin	1.47	Niger	3.21	Namibia	0.36
26	Burundi	1.25	Gambia. The	3.15	Benin	−0.09
27	Burkina Faso	1.17	Burundi	3.03	Togo	−0.11
28	Angola	0.78	Benin	3.00	Burundi	−0.56
29	Togo	0.76	Mozambique	2.97	Mali	−1.86
30	Mozambique	0.16	Burkina Faso	1.86	Angola	−1.94
31	Eswatini	−0.60	Togo	1.61	Cameroon	−2.34
32	Cameroon	−1.08	Cameroon	0.19	Equatorial Guinea	−2.59
33	Mali	−1.11	Mali	−0.35	Mozambique	−2.89
34	Equatorial Guinea	−1.60	Equatorial Guinea	−0.41	Cote d'Ivoire	−3.75
35	Cote d'Ivoire	−2.58	Cote d'Ivoire	−1.26	Eswatini	−4.72
36	Nigeria	−5.67	Lesotho	−3.05	Nigeria	−6.55
37	Chad	−6.02	Chad	−4.60	Chad	−7.42
38	Lesotho	−6.30	Nigeria	−4.76	Lesotho	−9.42

Source: Data used in health gap computation and ranking were obtained from the World Bank.

In terms of the internet component of ICT, Figure 2 shows that the average level of internet access penetration differs from country to country. Specifically, Morocco (34.65), Tunisia (29.01), Mauritius (28.87), South Africa (26.0), and Cabo Verde (24.1) are the top five countries with the highest average internet penetration. Whereas, Congo Republic (4.2), Malawi (3.7), Chad (2.1), Niger (1.6), and Burundi (1.5) were the last performing countries with respect to the proportion of the population with internet accessibility. Like mobile cellular subscription, an improvement was observed in 2018 for internet access, nevertheless, evidence from Table 2 shows that 22 countries of 38 countries under consideration had internet penetration below the 2018 average, while five countries (Congo Republic, Malawi, Chad, Niger, and Burundi) have below the 2000–2018 average value. Table 2 shows that the performance of the internet component of ICT is below that of mobile cellular subscription. These descriptive analysis results are consistent with David and Grobler (11) who reported that low internet penetration in Africa is a result of less competition, frequent government restriction, and interference, as well as the high cost of ICT infrastructures. Table 1 also shows that in 2018, 23 countries (61% of the total countries examined) had an ICT index below the 2018 average of 88.5, suggesting that ICT deployment in the African region is largely unevenly distributed.

### Health gap

**Figure 3:**
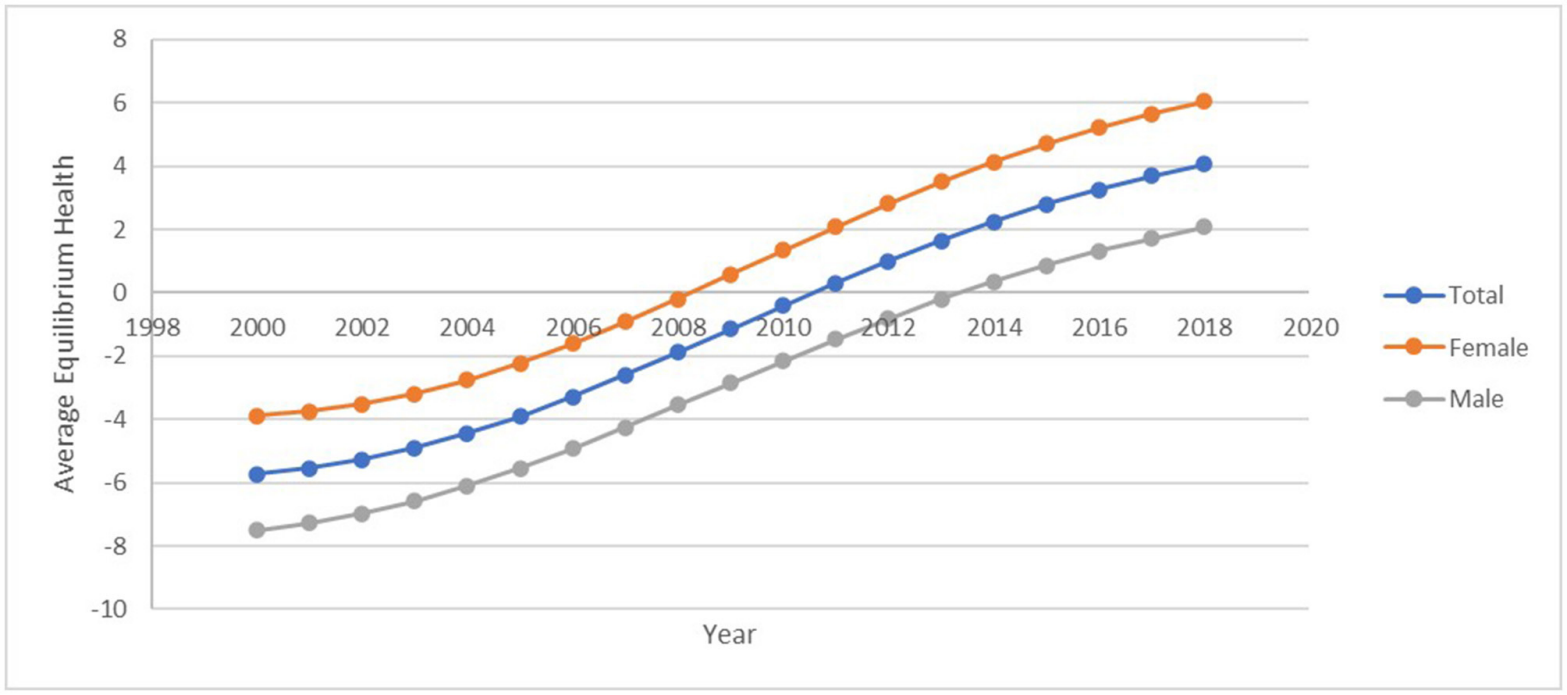
Average health gap –Africa. Source: Data used in computing the health gap was obtained from the World Bank.

During the 19-year period considered in this study, it can be seen from Figure 3 that there has been an impressive improvement in the size of the health outcome gap in the African region, as the gap moves from a negatively skewed one to a positively skewed one. The mean health outcome gap (desired life expectancy) was a 5.71-year deficit of target in 2000, it closed up reaching a gap of almost zero (0.37-year deficit) in 2010, and now stands at a 4.16-year surplus of the target in 2018. Despite the positive observation, there is still evidence of inequality with respect to gender. It is seen that the size of the health outcome gap differs between women and men; apparently, the mean health outcome gap for women was higher than for both men and the entire population throughout the period under consideration. The size of the health gap is negative and lesser for men in the year 2000, it is also positive but lower in 2018 relative to women. While the health outcome gap zero was attained for women in 2008, it took men extra 4 years to attain such height. Hossin (82) concisely stated that in almost all countries around the world, men consistently live shorter lives than women. This seems to be consistent with findings that though men are usually considered to be the stronger sex; nevertheless, when it comes to issues of health, men are observed to be weaker than women. This argument for a better health outcome for women unlike men is based on differences in health behavior life style. Women are more health conscious thus making them respond to health concerns (such as medical screening, check-ups, hygiene, and diets) and are less likely to take up risky health behaviors compared to men (83). The cumulated effect of these differences is a better life expectancy in favor of women.

**Figure 4:**
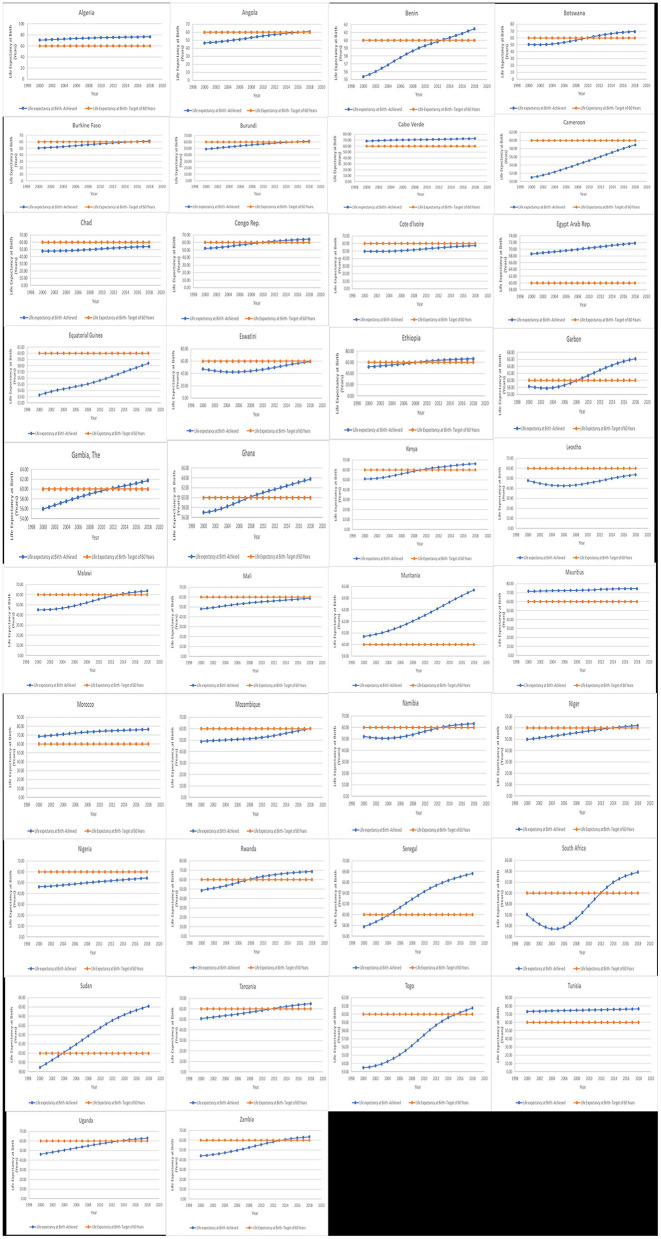
Trend in life expectancy at birth target (60 years) vs. life expectancy at birth achieved by the country. Source: Data used for the graph was obtained from the World Bank.

Figure 4 shows a clear presentation of the respective countries' performance with respect to the health gap. During the 19-year period, countries can be categorized differently considering their performance; these include (i) countries that had positively skewed health gap throughout the period under consideration (Algeria, Cabo Verde, Egypt, Mauritius Morocco and Tunisia) and (ii) countries that have a mixture of performance, improving from a negative performance to a positive one. It is, however, observed among this group of countries that their year of reaching a balanced target at 60 years differs. Countries in this category include Angola, Benin, Botswana, Burkina Faso, Burundi, Congo Republic, Eswatini, Ethiopia, Gabon, The Gambia, Ghana, Kenya, Malawi, Namibia, Niger, Rwanda, Senegal, South Africa, Sudan, Tanzania, Togo, Uganda, and Zambia. The third category of countries is those whose life expectancy at birth did not reach the target year of 60 throughout the period under consideration. These countries include Cameroon, Chad, Cote d'lvoire, Equatorial Guinea, Lesotho, Mali, and Nigeria.

A comparative descriptive analysis and ranking of health gap status among countries under examination as of 2018 are shown in Table 2. Table 2 shows that eight countries out of the 38 countries examined have negative health gap status. Meaning that these eight countries have not been able to meet desired life expectancy target of 60 since 2000 when the agreement was signed. The same pattern of gender difference is further observed in the year 2018, where only six countries did not meet the life expectancy at a birth target of 60 years for women and 13 countries for men. In terms of ranking, the top four countries with a positive and larger health gap are the North African countries (Algeria, Tunisia, and Morocco) and Mauritius in all three groups (health gap-total, women, and men) considered. Whereas, the consistently least-ranked countries are Nigeria, Lesotho, and Chad. It is surprising to see that a large economy such as Nigeria would be in the same lower cadre as smaller countries (Chad and Lesotho). The 2018 ranking of countries by their health gap status helps deduce that the size of the population does not absolutely matter in the issue of improving human health, rather it is the stability of the political institution, the commitment of the government to improving the health situation, and availability of health improving infrastructure that are most imperative.

### Summary of descriptive statistics of variables

Table 3 shows the summary statistics of 38 countries for the period 2000–2018. The results show that the mean value of the health gap variable is −1.003 years, with low records observed in Eswatini (−17.5 years) and Lesotho (−17.4 years) in 1993, Zambia (−16 years), and Malawi (−14.9 years) in 2001. The maximum value (16.7 years) which is observed in Algeria in 2011, is followed by other high records observed in Tunisia (16.5 years), Morocco (16.5 years), Mauritius (14.5 years), and Cabo Verde (12.8 years). In terms of ICT, the mean ICT index for the entire 38 countries is 43.518, with South Africa having the highest index (157.21) and Niger having the lowest value at 0.038. Considering the specific component of ICT (i.e., mobile phone subscriptions and internet access), it is estimated that a minimum value of the mobile cellular subscription of 0.0181 is recorded in Niger, while Botswana is estimated to have the largest number of mobile cellular subscriptions at 163.8. The average number of mobile cellular subscriptions is 50.051. The proportion of the population with access to the internet ranges from 0.0153% (minimum) in Ethiopia to 64.8% (maximum) in Morocco; the estimated average internet access is low at 11.493% for the period under consideration.

**Table 3:** Summary of descriptive statistics of variables.

**Variable**	**Obs**.	**Mean**	**Std. Dev**.	**Min**	**Max**
HG (Total)	722	−1.003	7.984	−17.482	16.693
ICTIndex	722	43.518	39.106	0.03	157.21
PuH	722	1.838	1.127	0.146	5.826
PrH	722	39.153	20.337	2.993	84.162
Y	722	$US 45.7 Billion	$US 85.0 Billion	$US 94.9 Billion	$US 466.9 Billion
U	722	41.065	18.203	8.246	89.37
E	722	66.202	11.928	44.78	89.65

Source: Computed by Authors.

The statistics show that public health expenditure as a proportion of general government expenditure ranges from a minimum of 0.146% attained by Cameroon in 2017 to a maximum record of 5.826% achieved by Lesotho in 2014; the mean public health expenditure as a proportion of general government expenditure for the 38 African countries is 1.838%. As regards private health expenditure, a minimum expenditure (i.e., out-of-pocket expenditure as a percentage of current health expenditure) of 2.993% is observed in Botswana in 2001, while the maximum (84.162%) was attained in Equatorial Guinea in 2007. However, the average private health expenditure among the countries under consideration for the period 2000–2018 is 39.153%. The mean GDP ranges from US$9.48 million in Cabo Verde to US$4.67bn in Nigeria, with an average of US$4.57bn. The proportion of the population residing in the urban areas is averaged at 41.065%, and it ranges from a maximum value of 89.37% attained by Gabon in 2018 to a minimum value of 8.246% reached by Burundi in 2000. With respect to labor participation, the maximum proportion is recorded in Tanzania in 2016 (89.65%) and the minimum in Algeria in 2015 (44.78%). The result of the computed correlation matrix among variables in form of their natural logarithm is shown in Table 4. It reveals that the health gap variable is positively correlated with the ICT index, public health expenditure, private health expenditure, and gross domestic product variables whereas it is negatively correlated with labor force participation and urbanization variables. Furthermore, considering the argument of Dohoo et al. (84) that a correlation value of 0.9 and above confirms the evidence of the existence of a multicollinearity problem, it can therefore be deduced that since the maximum correlation value seen in the first column (indicating the correlation between the dependent variable and explanatory factors) is 0.536, it can be concluded there is no possible multicollinearity among the variables in the model.

**Table 4:** Correlation matrix for life expectancy at birth-total.

**Variables**	**HG (Total)**	**lnICTIndedx**	**lngdp**	**lnhexp**	**lnphexp**	**lnu**	**lnl**
HG (Total)	1.000						
lnICTIndex	0.536	1.000					
lnY	0.295	0.252	1.000				
lnPuH	0.216	0.182	−0.004	1.000			
lnPrH	0.072	−0.183	0.154	−0.588	1.000		
lnU	−0.263	−0.026	−0.101	−0.118	−0.117	1.000	
lnE	−0.435	−0.323	−0.282	−0.144	−0.161	0.126	1.000

Source: Computed by Authors.

### Empirical estimation results

The results of Driscoll–Kraay standard errors regression estimation techniques are shown in Tables 5, 6 for the full sample and sub-samples of different sub-regional country groups. All the results are consistent and robust to the FGLS and PCSE that were also employed.

**Table 5:** Estimation results: Driscoll–Kraay standard errors - full sample.

	**HG (Total)**	**HG (Male)**	**HG (Female)**
lnICTIndex	2.272^*^ (0.338)	2.180^*^ (0.313)	2.354^*^ (0.363)
lnY	−0.014 (0.087)	0.082 (0.084)	−0.105 (0.089)
lnPuH	1.105^*^ (0.366)	0.922^**^ (0.388)	1.272^*^ (0.352)
lnPrH	−0.371^***^ (0.196)	−0.440^**^ (0.173)	−0.232 (0.228)
lnU	−1.192^*^ (0.119)	−1.125^*^ (0.111)	−1.276^*^ (0.129)
lnE	0.102 (1.523)	0.501 (1.488)	−0.471 (1.555)
North Africa	8.182^*^ (0.675)	8.540^*^ (0.613)	7.777^*^ (0.748)
Southern Africa	−6.0908 (0.297)	−6.705^*^ (0.259)	−5.316^*^ (0.336)
West Africa	−1.654^*^ (0.454)	−1.011^**^ (0.421)	−2.354^*^ (0.482)
Central Africa	−3.486^*^ (0.283)	−2.866^*^ (0.275)	−4.148^*^ (0.284)
cons	−11.137 (8.251)	−16.127^***^ (8.195)	−5.838 (8.287)
Number of Obs.	722	722	722
Number of groups	38	38	38
F	181300.77	209095.79	102674.34
Prob.>F	0.0000	0.0000	0.0000
R-Square	0.6303	0.6515	0.6129

* p < 0.01,

** p < 0.05,

*** p < 0.10. Standard errors are in parenthesis. Source: Computed by Authors.

**Table 6:** Estimation results: Driscoll–Kraay standard errors: by ICT components.

	**HG (Total)**	**HG (Male)**	**HG (Female)**
lnmobile	−0.554^*^ (0.154)	−0.439^**^ (0.169)	−0.669^*^ (0.145)
lnInternet	3.177^*^ (0.188)	2.941^*^ (0.169)	3.404^*^ (0.210)
lnY	−0.335^*^ (0.072)	−0.213^*^ (0.072)	−0.450^*^ (0.073)
lnPuH	0.557 (0.246)	0.418 (492)	0.683 (0.461)
lnPrH	−0.390^*^ (0.136)	−0.459^*^ (0.106)	−0.252 (0.173)
lnU	−1.022^*^ (0.158)	−0.969^*^ (0.146)	−1.093^*^ (0.173)
lnE	3.037^**^ (1.457)	3.202^**^ (1.363)	2.688^***^ (1.550)
North Africa	9.270^*^ (0.689)	9.547^*^ (0.623)	8.941^*^ (0.770)
Southern Africa	−5.777^*^ (0.160)	−6.416^*^ (0.149)	−4.979^*^ (0.186)
West Africa	−0.877^*^ (0.280)	−0.291 (0.243)	−1.522^*^ (0.321)
Central Africa	−2.239^*^ (0.456)	−1.713^*^ (0.444)	−2.809^*^ (0.456)
cons	−11.386 (7.155)	−16.338^**^ (7.120)	−6.121 (7.187)
Number of Obs.	722	722	722
Number of groups	38	38	38
F	84662.29	93668.79	94517.84
Prob.>F	0.0000	0.0000	0.0000
R–Square	0.6856	0.6117	0.6743

* p < 0.01,

** p < 0.05,

*** p < 0.10. Standard errors are in parenthesis. Source: Computed by Authors.

### The full sample with composite ICT Index

The result of the effect of ICT on the health gap based on the Driscoll–Kraay regression estimation is shown in Table 5. The coefficient for the ICT index variable is positive and statistically significant at *p* < 0.01 across the columns in Table 5, indicating that if all other variables are held constant, a 1% increase in the ICT index would lead to about a 0.022–0.024 point increase in the health gap, a gap that is positively skewed. This implies that with an increase in ICT development, the targeted health outcome of 60 years is met and exceeded. This finding is consistent with the *a priori* expectation of this study and previous empirical literature (60, 66, 68, 74) who all found a positive and statistically significant relationship between ICT and health outcomes and have demonstrated that ICT is a vital infrastructural facility that can enable the SSA region, in general, substantially exceed the life expectancy at birth at 60 years health target. ICT provides easy health information access, efficient sharing of patient health information among health service providers, and access to healthcare professionals often at a lower cost than for direct-person care in the office. It is also noticed from the result in Table 5 that public healthcare expenditure has a positive and significant (at 1 and 5% levels of significance) impact on the health gap variable. It can be inferred that a percentage increase in public healthcare expenditure will lead to an increase in the health gap by about 0.092–0.013 points if all other variables in the model are held constant. This result is consistent with the *a priori* expectation of this study in relation to the health capital model that public expenditure on health facilitates, programs, interventions, and necessary public health awareness promote positive health outcomes. This result is related to Nkemgha et al. (54) and Afroz et al. (72) who demonstrates the positive effect of public expenditure on the health outcome (life expectancy) examined in their respective study. This result implies that with an increase in public health expenditure, the targeted health outcome of 60 years is met and surpassed.

Contrary to the a priori expectation of this study, the Table 5 showed that the effect of private healthcare expenditure is negative and significant. When other variables are held constant, a 1% increase in private healthcare expenditure would lead to about a 0.037–0.039 point decline in the health gap, a gap that is skewed toward a negative direction. This implies that rather than moving toward the targeted life expectancy at birth, an increase in private healthcare spending would cause a shift from (or a falling behind) the targeted life expectancy at birth of 60 years. This is not unexpected because of the widespread reliance of the health system in the continent on private healthcare expenditure (57), or healthcare financing are from private sources and are majorly through out-of-pocket expenditure. Unfortunately, the utilization of private spending for healthcare amidst rising incidence of diseases, high healthcare costs, and a high prevalence of poverty in the majority of African countries that are in the low-income group encourages foregoing of healthcare needs, limits access to basic, critical, and high-quality healthcare services, and ultimately leads to undesirable health outcomes, which in this case is a reduction in the chances of meeting and exceeding the life expectancy target of 60 years.

The parameter of urbanization is negative and significant (*p* < 0.01) indicating that *ceteris paribus* a 1% increase in urbanization growth rate would lead to about a 0.012–0.013 point decline in the health gap. This decline is an indication that increased urbanization will lead to failure in meeting the expected life expectancy at birth target. This is not unexpected considering the high rate of rural–urban migration in the continent, and the observed unplanned rapid urbanization that deteriorates human health conditions through poor housing conditions, overcrowding, and poor environmental sanitation that stimulate the spread of diseases. The finding on this variable though consistent with Nathaniel and Khan (85), it is, however, contrary to the finding from Zhang et al. (86) and Mahalik et al. (77). A possible reason for this contrary finding is the heterogeneity in the preparedness for the level of urbanization across the region of the world, which was not taken into consideration in the two studies. Table 5 also shows negative and significant coefficients for all sub-regional variables except the NA sub-region. The coefficients for the NA variable across the columns are positive and statistically significant at *p* < 0.10, indicating that if all other variables are held constant, the NA sub-region can drive the African continent toward meeting and exceeding the targeted health outcome of life expectancy at birth of 60 years by about 0.07.8–0.08.2 points due to countries in the North African region of the continent. This could be a result of the level of wealth and a better healthcare system in the NA sub-region. In contrast, the parameters of SA, WA, and CA are negative and significant at 1, 5, and 10% levels of significance, respectively. These results imply that when other variables are held constant, SA, WA, and CA sub-regions reduce the potential of the African region to achieve the health outcome target of life expectancy at birth of 60 years. The prevalence of poverty and instability of government in these three sub-regions compared to the NA could be reasons for this result. Similarly, the difference or heterogeneity in economic performance in favor of the NA sub-region compared to other parts of Africa also contributes to other sub-regions' inability to promote the achievement of the internationally agreed health target.

### The full sample with components of ICT

Table 6 shows the results obtained from Driscoll–Kraay standard error regression estimation techniques where specific ICT components were taken into consideration. While the coefficients of mobile showed an unexpected negative relationship with the health gap variable, the internet variable on the other hand is revealed to have a positive and significant relationship with the health gap variable. Regarding control variables, it was found that economic performance, private healthcare expenditure, urbanization, SA, WA, and CA sub-regions of the continent all exhibited negative and statistically significant relationships with the health gap which is in line with the results obtained in Table 6. Succinctly, a 1% increase in mobile cellular subscription leads to a reduction in the health gap by about 0.044–0.067 points if other variables are held constant. That is an increase in mobile cellular subscriptions was more likely to decrease the chances of achieving the health outcome target of life expectancy at birth of 60 years. This implies that the mobile cellular subscription increase is not a stimulating factor in the pursuit of achieving The Declaration of Alma Ata, 1978 life expectancy at birth target in the African region in general. This result arises when multiple subscriptions by individuals leading to the increase, and size of phone ownership are not accounted for. An increase in mobile cellular subscriptions without an increase in ownership of phones will limit mobile phone usage for health purposes where privacy is needed and will also limit the effectiveness of mobile health intervention that would have aided the achievement of health outcomes (87). Furthermore, other plausible reasons for this result are the prevalence of poor network coverage and electricity problems in most poor underdeveloped African countries that limits phone usage for health purposes irrespective of the increase in subscription. The coefficient for the internet variable is positive and significant, indicating that a 1% increase in the percentage of people using the internet would lead to about a 0.029–0.034 point increase in the health gap. The increase here means that the life expectancy at birth targeted of 60 years is met and surpassed. This implies that the internet component of ICT is a vital infrastructure that can enhance health outcomes in the African region and without it, health policy goals or targets might not be achieved.

With respect to economic performance income and proxied by GDP, the negative coefficient implies that if all other variables are held constant, a 1% increase in the gross domestic product would lead to about a 0.021–0.045 point decline in the health gap, a gap that is skewed toward a negative direction. This suggests that rather than moving toward the targeted life expectancy at birth, an increase in income would cause a move away from (or a falling behind) the targeted life expectancy at birth of 60 years. It can, thus, be deduced that improved economic performance is not a stimulating factor in the pursuit of achieving The Declaration of Alma Ata, 1978 life expectancy at a birth target of 60 years in the African region. This result indicates that there has not been a trickle-down effect evidence of economic growth. The problems of poor funding of the health sector despite improving regional economic performance, given that many African countries have not achieved the agreed minimum health expenditure threshold declared in Abuja in 2001, the high prevalence of corruption in many African countries, and misappropriation of funds that could have been used for health infrastructure accumulation and promoting access to quality healthcare are possible explanations for this result.

### Robustness check- FGLS and PCSE

The robustness test of results obtained in Table 5 was examined by using other estimation techniques (FGLS and PCSE) which are shown in Table 7. It can be seen that the results are the same across the estimation techniques for each of the variables considered. In all the columns, the ICT index variable exhibited a positive and statistically significant relationship with the health gap variable when all other variables are held constant. This means that with ICT development, the gap between the achieved and the targeted health outcome goal would increase positively, indicating that the target will be attained and even exceeded.

**Table 7:** FGLS and PCSE (dependent variable: health gap).

	**HG (Total)**		**HG (Male)**		**HG (Female)**	
	**FGLS**	**PCSE**	**FGLS**	**PCSE**	**FGLS**	**PCSE**
lnICTIndedx	2.272^*^ (0.122)	2.272^*^ (0.195)	2.181^*^ (0.116)	2.181^*^ (0.178)	2.354^*^ (0.127)	2.354^*^ (0.212)
lnY	−0.014 (0.150)	−0.014 (0.058)	0.083 (0.143)	0.082 (0.054)	−0.104 (0.157)	−0.105^***^ (0.060)
lnPuH	1.105^*^ (0.414)	1.105^*^ (0.283)	0.923^**^ (0.395)	0.923^*^ (0.277)	1.272^*^ (0.435)	1.272^*^ (0.293)
lnPrH	−0.371 (0.422)	−0.371 (0.428)	−0.440 (0.402)	−0.440 (0.392)	−0.233 (0.442)	−0.233 (0.464)
lnU	−1.192^*^ (0.179)	−1.192^*^ (0.095)	−1.125^*^ (0.171)	−1.125^*^ (0.091)	−1.276^*^ (0.188)	−1.276^*^ (0.101)
lnE	0.102 (1.352)	0.102 (0.675)	0.501 (1.287)	0.501 (0.641)	−0.471 (1.416)	−0.471 (0.717)
North Africa	8.182^*^ (0.863)	8.182^*^ (0.474)	8.540^*^ (0.821)	8.540^*^ (0.419)	7.777^*^ (0.904)	7.777^*^ (0.535)
Southern Africa	−6.090^*^ (0.632)	−6.090^*^ (0.251)	−6.705^*^ (0.601)	−6.705^*^ (0.235)	−5.316^*^ (0.662)	−5.316^*^ (0.268)
West Africa	−1.654^*^ (0.609)	−1.654^*^ (0.324)	−1.011^***^ (0.580)	−1.011^*^ (0.308)	−2.355^*^ (0.638)	−2.354^*^ (0.340)
Central Africa	−3.487^*^ (0.759)	−3.487^*^ (0.212)	−2.866^*^ (0.723)	−2.866^*^ (0.216)	−4.148^*^ (0.795)	−4.148^*^ (0.206)
Constant	−11.137 (7.343)	−11.137^*^ (3.936)	−16.127^**^ (6.994)	−16.127^*^ (3.776)	−5.878 (7.693)	−5.838 (4.142)
Number of obs.	722	722	722	722	722	722
Number of groups	38	38	38	38	38	38
R-squared	-	0.6303	-	0.6515	-	0.6129
Wald Chi	1230.68	58883.15	1349.60	59265.87	1143.35	53925.05
Prob > chi2	0.0000	0.0000	0.0000	0.0000	0.0000	0.0000

* p < 0.01,

** p < 0.05,

*** p < 0.10. Standard errors are in parenthesis. Source: Computed by Authors.

### The full sample with composite ICT index by sub-region

Table A2 shows the results obtained from Driscoll–Kraay standard error estimations across sub-region to account for heterogeneity. The coefficients of the ICT index variable are positive and statistically significant (*p* < 0.01) across all the sub-regions. This shows that ICT is an enabler for the achievement of the health target in the continent. Again, it is interesting to note the sub-regional differences in the effect of employment on health target achievement. While the effect is significant and large for NA (28.035) and SA (13.482), it is negative and significant for WA (-7.925), CA (-3.744), and EA (-2.782), respectively. This implies that labor participation is not a stimulating factor in the pursuit of improving life expectancy in the WA, CA, and EA regions of Africa. This is apparently a reflection of the difference in the quality of jobs available across the continent. The quality of the jobs the labor force group is involved in, can enable, or inhibit access to quality health services that can improve life expectancy at birth. This further validates the imperativeness of the need for access to decent jobs in the African region.

## Conclusion and policy recommendations

By employing the Grossman Health Capital model, this study attempts to extend the existing literature on determinants of health by empirically investigating the impact of ICT on the health gap using estimation techniques suitable in cross-sectional and panel data of 38 African countries. In this study, while the health gap is measured by accounting for the difference between actual life expectancy at birth and life expectancy at 60 years as the benchmark for desired health outcome, the ICT index was computed using PCA from mobile subscription and internet access. The profiling of the ICT diffusion level shows that as of 2018, 61% of the country examined have ICT index values below the 2018 average index. This reflects that there is uneven ICT development in Africa. Eight countries out of the 38 countries examined have negative health gap status. Meaning that these eight countries have not been able to meet the life expectancy target of 60 years which is expected to have been achieved by 2000. Similarly, the number of countries having negative health gap status was found to differ by gender. Furthermore, this study confirms that increasing ICT increases health gap outcomes. This suggests that the probability of achieving and significantly exceeding health targets of life expectancy birth of 60 years (a health outcome target in The Declaration of Alma Ata (1978) which set a target of life expectancy >60 years by the year 2000), increase. Nevertheless, it is the internet component of ICT rather than the mobile that was found to be important in achieving the examined international health agreement. Public health expenditure and North Africa are other factors identified to aid the achievement of the health target, while urbanization, private health expenditure, Southern Africa, West Africa, and Central Africa were found to inhibit the achievement of the target. Thus, it can be said that though there appears to be a saturation in ICT development in Africa considering the decline in its growth rate of ICT components, it can be deduced that the health-enhancing potential of ICT through relevant policies can further be maximized. Specifically, measures and policies to reduce internet accessibility costs, improve internet coverage and connectivity for individuals and healthcare service-providing institutions, and enhance internet use skills for individuals and healthcare system workers must be deliberately put in place. Specific policies include but are not limited to declaring internet availability and access as a public good to promote access in several African countries. Similarly, incentives to internet service providers by the government can promote internet access to underserved areas and affordability. Policies that focus on promoting internet use competencies should be designed and executed. This study has its limitations. The study was limited to only 38 countries in Africa and was considered within static panel econometric estimation techniques. Thus, to better understand how ICT impact the achievement of international health, future studies might look at other developing region of the world such as Asia and from a dynamic panel perspective. Equally, other components of ICT (e.g., mobile–broadband network and international bandwidth usage) can be considered by future studies.

## Author's note

A health gap implies a difference/deviation between the achieved life expectancy at birth of 60 years and the targeted life expectancy at birth of 60 years.

## Data availability statement

Publicly available datasets were analyzed in this study. This data can be found at: https://data.worldbank.org/; https://www.itu.int/.

## Author contributions

All authors listed have made a substantial, direct, and intellectual contribution to the work and approved it for publication.

## Appendix

**Table A1:** Driscoll–Kraay standard errors by regions.

	**HG (North Africa)**	**HG (Southern Africa)**	**HG (West Africa)**	**HG (Central Africa)**	**HG (East Africa)**
lnICTIndedx	0.363^***^ (0.131)	3.863^*^ (0.553)	1.609^*^ (0.232)	1.554^**^ (0.370)	1.807^*^ (0.125)
Lngdp	1.652^*^ (0.269)	0.762^*^ (0.057)	−1.384^*^ (0.185)	0.096 (0.837)	0.728^**^ (0.238)
Lnhexp	5.864^*^ (0.436)	−0.394 (0.381)	4.861^*^ (0.328)	−0.129 (0.420)	−3.102^*^ (0.812)
Lnphexp	−1.779 (0.344)	0.888 (0.867)	−0.774^***^ (0.378)	−6.449^*^ (0.539)	−2.428 (1.780)
Lnurbgr	0.374 (206)	−5.059^*^ (0.505)	−0.009 (0.170)	1.392^*^ (0.280)	−0.996^***^ (0.409)
lnl	28.035^*^ (4.671)	13.482^*^ (3.273	−7.925^*^ (0.769)	−3.744^**^ (1.207)	−2.782 (2.833)
cons	−135.916^*^ (12.242)	−116.6684^*^ (14.604)	60.613^*^ (3.036)	37.603^**^ (5.176)	−5.155 (20.078)
Number of Obs.	95	190	209	95	133
Number of groups	5	9	11	6	7
F	381.84	162.02	889.23	207.98	331.70
Prob.>F	0.0000	0.0000	0.0000	0.0001	0.0000
R–Square	0.9284	0.6439	0.7235	0.8492	0.7406

*** p < 0.01,

** p < 0.05,

* p < 0.1. Standard errors are in parenthesis. Source: Computed by Authors (2022).

**Table A2:** Lists of countries in the sample.

**S/No**.	**Country**	**Region**	**S/No**.	**Country**	**Region**
1	Algeria	NA	20	Lesotho	SA
2	Angola	CA	21	Malawi	SA
3	Benin	WA	22	Mali	WA
4	Botswana	SA	23	Mauritania	NA
5	Burkina Faso	WA	24	Mauritius	SA
6	Burundi	EA	25	Morocco	NA
7	Cabo Verde	WA	26	Mozambique	SA
8	Cameroon	CA	27	Namibia	SA
9	Chad	CA	28	Niger	WA
10	Congo. Rep.	EA	29	Nigeria	WA
11	Cote d'Ivoire	WA	30	Rwanda	EA
12	Egypt. Arab Rep.	NA	31	Senegal	WA
13	Equatorial Guinea	CA	32	South Africa	SA
14	Eswatini	SA	33	Sudan	EA
15	Ethiopia	CA	34	Tanzania	EA
16	Gabon	CA	35	Togo	WA
17	Gambia. The	WA	36	Tunisia	NA
18	Ghana	WA	37	Uganda	EA
19	Kenya	EA	38	Zambia	SA

NA, North Africa; CA, Central Africa; EA, East Africa; SA, Southern Africa; WA, West Africa.
